# 1′-Methyl-4′-phenyl­dispiro­[chromane-3,3′-pyrrolidine-2′,3′′-indoline]-2,2′′-dione

**DOI:** 10.1107/S1600536812008288

**Published:** 2012-02-29

**Authors:** Jagadeesan Ganapathy, Kannan Damodharan, Bakthadoss Manickam, Aravindhan Sanmargam, Gunasekaran Krishnasamy

**Affiliations:** a Department of Physics, Presidency College, Chennai 600 005, India; b Department of Organic Chemistry, University of Madras, Guindy Campus, Chennai 600 025, India; c CAS in Crystallography and Biophysics, University of Madras, Guindy Campus, Chennai 600 025, India

## Abstract

In the title compound, C_26_H_22_N_2_O_3_, the pyrrolidine ring adopts an envelope conformation with the N atom as the flap. In the crystal, pairs of centrosymmetrically related mol­ecules are linked into dimers by N—H⋯O hydrogen bonds. In addition, there are C—H⋯O hydrogen bonds.

## Related literature

For a related structure, see: Gangadharan *et al.* (2010 ▶).
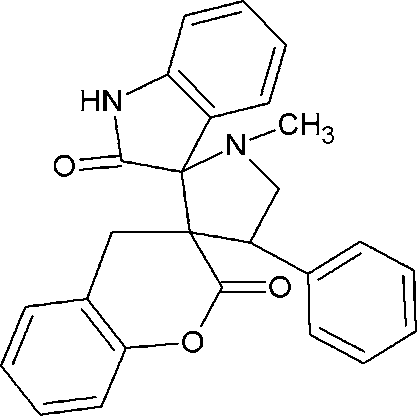


## Experimental

### 

#### Crystal data

C_26_H_22_N_2_O_3_
*M* *_r_* = 410.46
Triclinic, 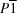

*a* = 8.9280 (4) Å
*b* = 10.0923 (4) Å
*c* = 11.9044 (5) Å
α = 95.027 (1)°
β = 93.172 (1)°
γ = 98.991 (2)°
*V* = 1052.78 (8) Å^3^
*Z* = 2
Mo *K*α radiation
μ = 0.09 mm^−1^
*T* = 293 K
0.25 × 0.20 × 0.20 mm

#### Data collection

Bruke Kappa APEXII CCD diffractometer
Absorption correction: multi-scan (*SADABS*; Bruker, 2004 ▶) *T*_min_ = 0.979, *T*_max_ = 0.983
28847 measured reflections
7433 independent reflections
4947 reflections with *I* > 2σ(*I*)
*R*_int_ = 0.028

#### Refinement

*R*[*F* ^2^ > 2σ(*F* ^2^)] = 0.063
*wR*(*F* ^2^) = 0.190
*S* = 1.05
7433 reflections
289 parameters
H atoms treated by a mixture of independent and constrained refinement
Δρ_max_ = 0.45 e Å^−3^
Δρ_min_ = −0.40 e Å^−3^

### 

Data collection: *APEX2* (Bruker, 2004 ▶); cell refinement: *APEX2* and *SAINT* (Bruker, 2004 ▶); data reduction: *SAINT* and *XPREP* (Bruker, 2004 ▶); program(s) used to solve structure: *SHELXS97* (Sheldrick, 2008 ▶); program(s) used to refine structure: *SHELXL97* (Sheldrick, 2008 ▶); molecular graphics: *ORTEP-3 for Windows* (Farrugia, 1997 ▶); software used to prepare material for publication: *PLATON* (Spek, 2009 ▶).

## Supplementary Material

Crystal structure: contains datablock(s) I, global. DOI: 10.1107/S1600536812008288/bt5810sup1.cif

Structure factors: contains datablock(s) I. DOI: 10.1107/S1600536812008288/bt5810Isup2.hkl

Additional supplementary materials:  crystallographic information; 3D view; checkCIF report

## Figures and Tables

**Table 1:** Hydrogen-bond geometry (Å, °)

*D*—H⋯*A*	*D*—H	H⋯*A*	*D*⋯*A*	*D*—H⋯*A*
N2—H2⋯O1^i^	0.90 (2)	1.94 (2)	2.8362 (16)	169 (2)
C21—H21⋯O2^ii^	0.93	2.53	3.261 (3)	136 (0)

Symmetry codes: (i) 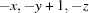; (ii) 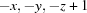.

**Fig. 1.**
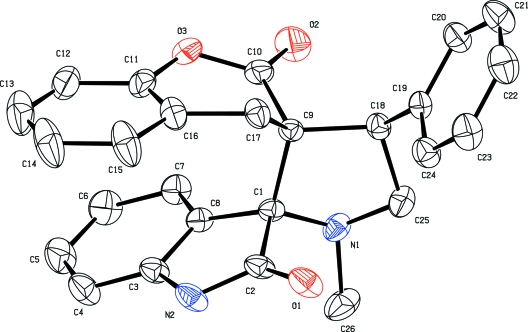
The molecular structure of the title compound showing 30% probability displacement ellipsoids for non-H atoms

**Fig. 2.**
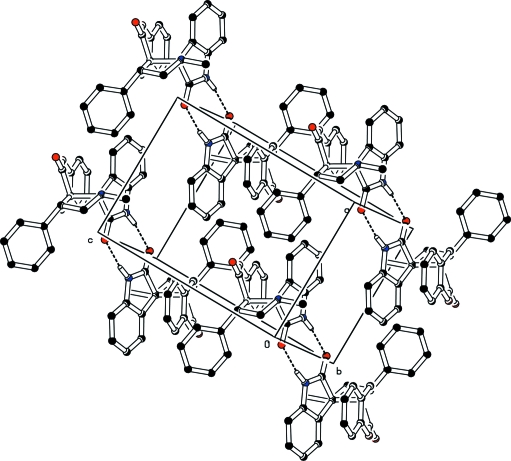
Crystal packing diagram. H atoms not involved in hydrogen bonding (dashed lines) have been omitted for clarity.

## supplementary crystallographic information

### Comment

Substituted pyrrolidine compounds have gained much importance since they are the basic structural elements of many alkaloids and pharmacologically active compounds while molecules with the indole moiety posses anti-inflammatory and antibacterial properties. In view of their importance, the crystal structure determination of the title compound was carried out and the results are presented herein. The molecular structure of the title compound is shown in Fig. 1. In the molecule, the oxindole moiety and phenyl ring are almost coplanar with the pyrrolidine ring attached to C1 and C18 respectively. Dihedral angle (C9—C18—C19—C24) formed between pyrrolidine ring and benzene ring is 71.4 (2) ° and the dihedral angle (C2—C1—N1—C26) between oxindole moiety and benzene ring is 48.8 (2) °. Pyrrolidine ring adopts envelope conformation (Gangadharan *et al.*, 2010) and the benzopyran ring adopts half chair conformation. In the crystal, pairs of centrosymmetrically related molecules are linked into dimers by C21—H21···O2, N2—H2···O1 hydrogen bonds (Fig. 2).

### Experimental

A mixture of (*E*)-3-benzylidinechroman-2-one (0.118 g, 0.5 mmol), isatin (0.080 g, 0.55 mmol) and *N*-methylglycine (0.025 g, 0.55 mmol) in toluene (5 ml) was refluxed. After completion of the reaction as indicated by TLC, the reaction mixture was concentrated and the resulting crude mass was diluted with water (10 ml) and extracted with ethyl acetate (3 *x* 10 ml). The combined organic layer was concentrated under reduced pressure. The crude mass was purified by column chromatography on silica gel (Acme 100–200 mesh), using ethylacetate: hexanes (2: 8) to afford the 1'-methyl-4'-phenyl-1",2,2",4-tetrahydro dispiro[1-benzopyran-3,3'-pyrrolidine-2',3''-indole]-2, 2''-dione as a colorless solid.

### Refinement

The H atoms H18, H2 were isotropically refined. All other H atoms were positioned geometrically and were treated as riding on their parent atoms, with C—H distances of 0.93–0.97 Å and *U*_iso_(H)=1.2*U*_eq_(C) or *U*_iso_(H)=1.5*U*_eq_(C_methyl_).

### Crystal data

**Table:**

C_26_H_22_N_2_O_3_	*Z* = 2
*M**_r_* = 410.46	*F*(000) = 432
Triclinic, *P*1	*D*_x_ = 1.295 Mg m^−^^3^
Hall symbol: -P 1	Mo *K*α radiation, λ = 0.71073 Å
*a* = 8.9280 (4) Å	Cell parameters from 8834 reflections
*b* = 10.0923 (4) Å	θ = 2.1–31.2°
*c* = 11.9044 (5) Å	µ = 0.09 mm^−^^1^
α = 95.027 (1)°	*T* = 293 K
β = 93.172 (1)°	Block, colourless
γ = 98.991 (2)°	0.25 × 0.20 × 0.20 mm
*V* = 1052.78 (8) Å^3^	

### Data collection

**Table:**

Bruke Kappa APEXII CCD diffractometer	7433 independent reflections
Radiation source: fine-focus sealed tube	4947 reflections with *I* > 2σ(*I*)
Graphite monochromator	*R*_int_ = 0.028
ω and φ scan	θ_max_ = 32.3°, θ_min_ = 2.1°
Absorption correction: multi-scan (*SADABS*; Bruker, 2004)	*h* = −13→13
*T*_min_ = 0.979, *T*_max_ = 0.983	*k* = −15→15
28847 measured reflections	*l* = −17→17

### Refinement

**Table:**

Refinement on *F*^2^	Primary atom site location: structure-invariant direct methods
Least-squares matrix: full	Secondary atom site location: difference Fourier map
*R*[*F*^2^ > 2σ(*F*^2^)] = 0.063	Hydrogen site location: inferred from neighbouring sites
*wR*(*F*^2^) = 0.190	H atoms treated by a mixture of independent and constrained refinement
*S* = 1.05	*w* = 1/[σ^2^(*F*_o_^2^) + (0.0895*P*)^2^ + 0.2365*P*] where *P* = (*F*_o_^2^ + 2*F*_c_^2^)/3
7433 reflections	(Δ/σ)_max_ < 0.001
289 parameters	Δρ_max_ = 0.45 e Å^−^^3^
0 restraints	Δρ_min_ = −0.40 e Å^−^^3^

### Special details

**Table:**

Geometry. All e.s.d.'s (except the e.s.d. in the dihedral angle between two l.s. planes) are estimated using the full covariance matrix. The cell e.s.d.'s are taken into account individually in the estimation of e.s.d.'s in distances, angles and torsion angles; correlations between e.s.d.'s in cell parameters are only used when they are defined by crystal symmetry. An approximate (isotropic) treatment of cell e.s.d.'s is used for estimating e.s.d.'s involving l.s. planes.
Refinement. Refinement of *F*^2^ against ALL reflections. The weighted *R*-factor *wR* and goodness of fit *S* are based on *F*^2^, conventional *R*-factors *R* are based on *F*, with *F* set to zero for negative *F*^2^. The threshold expression of *F*^2^ > σ(*F*^2^) is used only for calculating *R*-factors(gt) *etc*. and is not relevant to the choice of reflections for refinement. *R*-factors based on *F*^2^ are statistically about twice as large as those based on *F*, and *R*- factors based on ALL data will be even larger.

### Fractional atomic coordinates and isotropic or equivalent isotropic displacement parameters (Å^2^)

**Table:**

	*x*	*y*	*z*	*U*_iso_*/*U*_eq_	
C1	0.18688 (14)	0.30903 (14)	0.18304 (10)	0.0327 (3)	
C2	0.09250 (16)	0.38274 (17)	0.10248 (12)	0.0418 (3)	
C3	0.31829 (16)	0.52072 (17)	0.14566 (12)	0.0419 (3)	
C4	0.4325 (2)	0.6297 (2)	0.14951 (17)	0.0582 (5)	
H4	0.4204	0.7049	0.1122	0.070*	
C5	0.5666 (2)	0.6226 (2)	0.21141 (18)	0.0635 (5)	
H5	0.6454	0.6955	0.2173	0.076*	
C6	0.58552 (19)	0.5102 (2)	0.26430 (17)	0.0587 (5)	
H6	0.6775	0.5074	0.3040	0.070*	
C7	0.46918 (17)	0.40086 (17)	0.25926 (13)	0.0452 (3)	
H7	0.4828	0.3241	0.2940	0.054*	
C8	0.33290 (15)	0.40837 (15)	0.20164 (11)	0.0351 (3)	
C9	0.09670 (14)	0.27119 (13)	0.28914 (10)	0.0317 (3)	
C10	0.21315 (19)	0.27681 (17)	0.39002 (12)	0.0461 (4)	
C11	0.2129 (2)	0.51265 (18)	0.42028 (13)	0.0525 (4)	
C12	0.3071 (3)	0.6340 (2)	0.45399 (17)	0.0697 (6)	
H12	0.3998	0.6361	0.4943	0.084*	
C13	0.2604 (3)	0.7499 (2)	0.4267 (2)	0.0862 (8)	
H13	0.3221	0.8322	0.4486	0.103*	
C14	0.1256 (3)	0.7471 (2)	0.3680 (3)	0.0917 (9)	
H14	0.0953	0.8274	0.3507	0.110*	
C15	0.0312 (2)	0.62413 (19)	0.3332 (2)	0.0744 (7)	
H15	−0.0610	0.6228	0.2923	0.089*	
C16	0.0758 (2)	0.50551 (16)	0.35981 (15)	0.0513 (4)	
C17	−0.01337 (17)	0.36729 (14)	0.32385 (14)	0.0421 (3)	
H17A	−0.0853	0.3718	0.2607	0.051*	
H17B	−0.0699	0.3344	0.3858	0.051*	
C18	0.02149 (16)	0.12015 (14)	0.25787 (12)	0.0367 (3)	
C19	−0.14732 (17)	0.08374 (14)	0.26735 (12)	0.0383 (3)	
C20	−0.2008 (2)	0.00358 (17)	0.34999 (15)	0.0493 (4)	
H20	−0.1316	−0.0238	0.4008	0.059*	
C21	−0.3542 (2)	−0.0364 (2)	0.35854 (19)	0.0636 (5)	
H21	−0.3876	−0.0909	0.4142	0.076*	
C22	−0.4578 (2)	0.0044 (2)	0.2848 (2)	0.0648 (5)	
H22	−0.5615	−0.0213	0.2909	0.078*	
C23	−0.4073 (2)	0.08315 (19)	0.20232 (18)	0.0577 (4)	
H23	−0.4772	0.1106	0.1522	0.069*	
C24	−0.25367 (19)	0.12229 (17)	0.19277 (14)	0.0484 (4)	
H24	−0.2212	0.1750	0.1358	0.058*	
C25	0.07155 (18)	0.08433 (17)	0.14028 (14)	0.0472 (4)	
H25A	0.0900	−0.0081	0.1317	0.057*	
H25B	−0.0051	0.0957	0.0825	0.057*	
C26	0.2647 (2)	0.1770 (2)	0.01946 (15)	0.0658 (5)	
H26A	0.2859	0.0889	−0.0045	0.099*	
H26B	0.3558	0.2419	0.0201	0.099*	
H26C	0.1881	0.1996	−0.0319	0.099*	
N1	0.21084 (14)	0.17778 (14)	0.13261 (10)	0.0428 (3)	
N2	0.17482 (15)	0.50215 (16)	0.08736 (12)	0.0505 (4)	
O1	−0.03426 (13)	0.33760 (14)	0.05740 (10)	0.0572 (3)	
O2	0.26613 (17)	0.18314 (15)	0.41917 (12)	0.0730 (4)	
O3	0.26132 (16)	0.39709 (13)	0.45070 (9)	0.0581 (3)	
H18	0.073 (2)	0.0690 (19)	0.3078 (16)	0.053 (5)*	
H2	0.141 (3)	0.561 (3)	0.043 (2)	0.082 (7)*	

### Atomic displacement parameters (Å^2^)

**Table:**

	*U*^11^	*U*^22^	*U*^33^	*U*^12^	*U*^13^	*U*^23^
C1	0.0289 (6)	0.0406 (7)	0.0290 (5)	0.0065 (5)	−0.0015 (4)	0.0062 (5)
C2	0.0331 (7)	0.0558 (9)	0.0372 (6)	0.0032 (6)	−0.0030 (5)	0.0192 (6)
C3	0.0322 (7)	0.0527 (9)	0.0410 (7)	0.0021 (6)	0.0025 (5)	0.0139 (6)
C4	0.0464 (9)	0.0589 (11)	0.0683 (11)	−0.0048 (8)	0.0023 (8)	0.0254 (9)
C5	0.0396 (9)	0.0687 (12)	0.0760 (12)	−0.0117 (8)	0.0004 (8)	0.0120 (10)
C6	0.0315 (7)	0.0757 (13)	0.0653 (10)	0.0010 (8)	−0.0071 (7)	0.0068 (9)
C7	0.0328 (7)	0.0540 (9)	0.0485 (8)	0.0081 (6)	−0.0040 (6)	0.0053 (7)
C8	0.0283 (6)	0.0447 (7)	0.0324 (5)	0.0056 (5)	0.0012 (4)	0.0047 (5)
C9	0.0321 (6)	0.0341 (6)	0.0294 (5)	0.0060 (5)	0.0000 (4)	0.0064 (4)
C10	0.0500 (9)	0.0525 (9)	0.0335 (6)	0.0004 (7)	−0.0050 (6)	0.0097 (6)
C11	0.0573 (10)	0.0521 (9)	0.0420 (7)	−0.0065 (8)	0.0170 (7)	−0.0106 (7)
C12	0.0730 (13)	0.0658 (13)	0.0579 (10)	−0.0195 (10)	0.0225 (9)	−0.0181 (9)
C13	0.0796 (16)	0.0548 (12)	0.1143 (19)	−0.0182 (11)	0.0515 (15)	−0.0220 (12)
C14	0.0894 (18)	0.0363 (10)	0.153 (3)	0.0090 (10)	0.0573 (18)	0.0030 (12)
C15	0.0570 (11)	0.0395 (9)	0.130 (2)	0.0092 (8)	0.0375 (12)	0.0069 (11)
C16	0.0527 (9)	0.0362 (8)	0.0659 (10)	0.0049 (7)	0.0294 (8)	−0.0011 (7)
C17	0.0398 (7)	0.0343 (7)	0.0533 (8)	0.0055 (6)	0.0146 (6)	0.0039 (6)
C18	0.0383 (7)	0.0314 (6)	0.0409 (6)	0.0068 (5)	−0.0008 (5)	0.0068 (5)
C19	0.0410 (7)	0.0289 (6)	0.0438 (7)	0.0033 (5)	0.0020 (5)	0.0024 (5)
C20	0.0507 (9)	0.0420 (8)	0.0550 (9)	0.0032 (7)	0.0026 (7)	0.0129 (7)
C21	0.0551 (11)	0.0607 (11)	0.0745 (12)	−0.0034 (9)	0.0131 (9)	0.0216 (9)
C22	0.0429 (9)	0.0596 (11)	0.0901 (14)	−0.0007 (8)	0.0113 (9)	0.0090 (10)
C23	0.0426 (9)	0.0532 (10)	0.0763 (12)	0.0074 (7)	−0.0062 (8)	0.0083 (9)
C24	0.0447 (8)	0.0454 (8)	0.0547 (8)	0.0045 (7)	−0.0024 (7)	0.0121 (7)
C25	0.0454 (8)	0.0435 (8)	0.0495 (8)	0.0046 (6)	0.0030 (6)	−0.0083 (6)
C26	0.0545 (10)	0.0891 (15)	0.0478 (9)	0.0027 (10)	0.0143 (8)	−0.0172 (9)
N1	0.0386 (6)	0.0483 (7)	0.0398 (6)	0.0072 (5)	0.0044 (5)	−0.0064 (5)
N2	0.0375 (7)	0.0602 (8)	0.0551 (7)	0.0006 (6)	−0.0069 (5)	0.0322 (7)
O1	0.0380 (6)	0.0728 (8)	0.0591 (7)	−0.0045 (5)	−0.0165 (5)	0.0340 (6)
O2	0.0793 (10)	0.0703 (9)	0.0686 (8)	0.0103 (7)	−0.0305 (7)	0.0277 (7)
O3	0.0700 (8)	0.0631 (8)	0.0334 (5)	−0.0065 (6)	−0.0066 (5)	−0.0011 (5)

### Geometric parameters (Å, º)

**Table:**

C1—N1	1.4576 (19)	C14—C15	1.403 (3)
C1—C8	1.5081 (19)	C14—H14	0.9300
C1—C2	1.5538 (18)	C15—C16	1.376 (3)
C1—C9	1.5780 (17)	C15—H15	0.9300
C2—O1	1.2269 (17)	C16—C17	1.508 (2)
C2—N2	1.342 (2)	C17—H17A	0.9700
C3—C4	1.374 (2)	C17—H17B	0.9700
C3—C8	1.384 (2)	C18—C19	1.507 (2)
C3—N2	1.4004 (18)	C18—C25	1.524 (2)
C4—C5	1.385 (3)	C18—H18	0.963 (19)
C4—H4	0.9300	C19—C20	1.386 (2)
C5—C6	1.373 (3)	C19—C24	1.387 (2)
C5—H5	0.9300	C20—C21	1.378 (3)
C6—C7	1.387 (2)	C20—H20	0.9300
C6—H6	0.9300	C21—C22	1.374 (3)
C7—C8	1.3791 (18)	C21—H21	0.9300
C7—H7	0.9300	C22—C23	1.369 (3)
C9—C17	1.5329 (19)	C22—H22	0.9300
C9—C10	1.5355 (18)	C23—C24	1.381 (2)
C9—C18	1.5703 (19)	C23—H23	0.9300
C10—O2	1.190 (2)	C24—H24	0.9300
C10—O3	1.352 (2)	C25—N1	1.451 (2)
C11—C16	1.374 (3)	C25—H25A	0.9700
C11—O3	1.376 (2)	C25—H25B	0.9700
C11—C12	1.386 (3)	C26—N1	1.455 (2)
C12—C13	1.361 (4)	C26—H26A	0.9600
C12—H12	0.9300	C26—H26B	0.9600
C13—C14	1.352 (4)	C26—H26C	0.9600
C13—H13	0.9300	N2—H2	0.90 (2)
			
N1—C1—C8	112.08 (11)	C11—C16—C17	117.49 (15)
N1—C1—C2	112.81 (11)	C15—C16—C17	124.38 (19)
C8—C1—C2	100.71 (11)	C16—C17—C9	109.17 (12)
N1—C1—C9	102.40 (10)	C16—C17—H17A	109.8
C8—C1—C9	118.45 (10)	C9—C17—H17A	109.8
C2—C1—C9	110.83 (10)	C16—C17—H17B	109.8
O1—C2—N2	125.61 (13)	C9—C17—H17B	109.8
O1—C2—C1	125.71 (13)	H17A—C17—H17B	108.3
N2—C2—C1	108.65 (11)	C19—C18—C25	113.59 (12)
C4—C3—C8	122.53 (14)	C19—C18—C9	117.93 (11)
C4—C3—N2	128.00 (15)	C25—C18—C9	104.50 (11)
C8—C3—N2	109.47 (13)	C19—C18—H18	108.4 (11)
C3—C4—C5	117.08 (17)	C25—C18—H18	105.9 (11)
C3—C4—H4	121.5	C9—C18—H18	105.6 (11)
C5—C4—H4	121.5	C20—C19—C24	117.72 (15)
C6—C5—C4	121.37 (17)	C20—C19—C18	119.68 (13)
C6—C5—H5	119.3	C24—C19—C18	122.54 (13)
C4—C5—H5	119.3	C21—C20—C19	121.44 (16)
C5—C6—C7	120.81 (16)	C21—C20—H20	119.3
C5—C6—H6	119.6	C19—C20—H20	119.3
C7—C6—H6	119.6	C22—C21—C20	119.96 (17)
C8—C7—C6	118.55 (15)	C22—C21—H21	120.0
C8—C7—H7	120.7	C20—C21—H21	120.0
C6—C7—H7	120.7	C23—C22—C21	119.50 (17)
C7—C8—C3	119.56 (13)	C23—C22—H22	120.2
C7—C8—C1	130.97 (13)	C21—C22—H22	120.2
C3—C8—C1	109.44 (11)	C22—C23—C24	120.71 (17)
C17—C9—C10	106.84 (12)	C22—C23—H23	119.6
C17—C9—C18	115.14 (11)	C24—C23—H23	119.6
C10—C9—C18	108.11 (11)	C23—C24—C19	120.66 (16)
C17—C9—C1	114.41 (10)	C23—C24—H24	119.7
C10—C9—C1	107.83 (11)	C19—C24—H24	119.7
C18—C9—C1	104.19 (10)	N1—C25—C18	104.63 (12)
O2—C10—O3	116.72 (14)	N1—C25—H25A	110.8
O2—C10—C9	125.09 (15)	C18—C25—H25A	110.8
O3—C10—C9	118.19 (14)	N1—C25—H25B	110.8
C16—C11—O3	120.30 (15)	C18—C25—H25B	110.8
C16—C11—C12	122.4 (2)	H25A—C25—H25B	108.9
O3—C11—C12	117.26 (19)	N1—C26—H26A	109.5
C13—C12—C11	118.4 (2)	N1—C26—H26B	109.5
C13—C12—H12	120.8	H26A—C26—H26B	109.5
C11—C12—H12	120.8	N1—C26—H26C	109.5
C14—C13—C12	121.0 (2)	H26A—C26—H26C	109.5
C14—C13—H13	119.5	H26B—C26—H26C	109.5
C12—C13—H13	119.5	C25—N1—C26	113.77 (13)
C13—C14—C15	120.6 (2)	C25—N1—C1	107.07 (11)
C13—C14—H14	119.7	C26—N1—C1	115.04 (14)
C15—C14—H14	119.7	C2—N2—C3	111.66 (12)
C16—C15—C14	119.6 (2)	C2—N2—H2	123.0 (16)
C16—C15—H15	120.2	C3—N2—H2	125.4 (16)
C14—C15—H15	120.2	C10—O3—C11	121.15 (13)
C11—C16—C15	118.10 (18)		
			
N1—C1—C2—O1	55.8 (2)	O3—C11—C16—C17	−3.1 (2)
C8—C1—C2—O1	175.48 (16)	C12—C11—C16—C17	177.50 (15)
C9—C1—C2—O1	−58.3 (2)	C14—C15—C16—C11	0.0 (3)
N1—C1—C2—N2	−122.10 (15)	C14—C15—C16—C17	−177.93 (19)
C8—C1—C2—N2	−2.45 (16)	C11—C16—C17—C9	−38.46 (19)
C9—C1—C2—N2	123.75 (14)	C15—C16—C17—C9	139.47 (17)
C8—C3—C4—C5	0.8 (3)	C10—C9—C17—C16	55.64 (15)
N2—C3—C4—C5	−178.43 (18)	C18—C9—C17—C16	175.70 (12)
C3—C4—C5—C6	1.5 (3)	C1—C9—C17—C16	−63.61 (16)
C4—C5—C6—C7	−1.4 (3)	C17—C9—C18—C19	−1.43 (17)
C5—C6—C7—C8	−1.2 (3)	C10—C9—C18—C19	117.93 (13)
C6—C7—C8—C3	3.4 (2)	C1—C9—C18—C19	−127.55 (12)
C6—C7—C8—C1	−178.90 (15)	C17—C9—C18—C25	125.80 (13)
C4—C3—C8—C7	−3.3 (2)	C10—C9—C18—C25	−114.84 (13)
N2—C3—C8—C7	176.04 (14)	C1—C9—C18—C25	−0.32 (13)
C4—C3—C8—C1	178.51 (16)	C25—C18—C19—C20	125.64 (16)
N2—C3—C8—C1	−2.10 (17)	C9—C18—C19—C20	−111.63 (15)
N1—C1—C8—C7	−55.00 (19)	C25—C18—C19—C24	−51.34 (19)
C2—C1—C8—C7	−175.16 (15)	C9—C18—C19—C24	71.39 (18)
C9—C1—C8—C7	63.9 (2)	C24—C19—C20—C21	−0.2 (3)
N1—C1—C8—C3	122.86 (13)	C18—C19—C20—C21	−177.37 (16)
C2—C1—C8—C3	2.69 (14)	C19—C20—C21—C22	−0.6 (3)
C9—C1—C8—C3	−118.24 (13)	C20—C21—C22—C23	0.9 (3)
N1—C1—C9—C17	−149.91 (11)	C21—C22—C23—C24	−0.2 (3)
C8—C1—C9—C17	86.25 (14)	C22—C23—C24—C19	−0.7 (3)
C2—C1—C9—C17	−29.36 (16)	C20—C19—C24—C23	0.9 (2)
N1—C1—C9—C10	91.39 (12)	C18—C19—C24—C23	177.93 (15)
C8—C1—C9—C10	−32.45 (16)	C19—C18—C25—N1	154.07 (12)
C2—C1—C9—C10	−148.06 (12)	C9—C18—C25—N1	24.21 (15)
N1—C1—C9—C18	−23.33 (12)	C18—C25—N1—C26	−169.91 (15)
C8—C1—C9—C18	−147.17 (12)	C18—C25—N1—C1	−41.67 (15)
C2—C1—C9—C18	97.22 (12)	C8—C1—N1—C25	168.45 (11)
C17—C9—C10—O2	142.82 (18)	C2—C1—N1—C25	−78.71 (14)
C18—C9—C10—O2	18.3 (2)	C9—C1—N1—C25	40.46 (13)
C1—C9—C10—O2	−93.76 (19)	C8—C1—N1—C26	−64.05 (15)
C17—C9—C10—O3	−37.78 (17)	C2—C1—N1—C26	48.79 (17)
C18—C9—C10—O3	−162.25 (13)	C9—C1—N1—C26	167.96 (12)
C1—C9—C10—O3	85.65 (16)	O1—C2—N2—C3	−176.54 (17)
C16—C11—C12—C13	0.6 (3)	C1—C2—N2—C3	1.40 (19)
O3—C11—C12—C13	−178.83 (17)	C4—C3—N2—C2	179.75 (18)
C11—C12—C13—C14	0.0 (3)	C8—C3—N2—C2	0.4 (2)
C12—C13—C14—C15	−0.5 (4)	O2—C10—O3—C11	176.59 (16)
C13—C14—C15—C16	0.6 (4)	C9—C10—O3—C11	−2.9 (2)
O3—C11—C16—C15	178.85 (16)	C16—C11—O3—C10	26.0 (2)
C12—C11—C16—C15	−0.6 (3)	C12—C11—O3—C10	−154.60 (15)

### Hydrogen-bond geometry (Å, º)

**Table:**

*D*—H···*A*	*D*—H	H···*A*	*D*···*A*	*D*—H···*A*
N2—H2···O1^i^	0.90 (2)	1.94 (2)	2.8362 (16)	169 (2)
C21—H21···O2^ii^	0.93	2.53	3.261 (3)	136 (0)

Symmetry codes: (i) −*x*, −*y*+1, −*z*; (ii) −*x*, −*y*, −*z*+1.
